# Selective Photothermal Eradication of Glioblastoma Cells Coexisting with Astrocytes by Anti-EGFR-Coated Raman Tags

**DOI:** 10.1021/acsabm.4c01986

**Published:** 2025-03-18

**Authors:** Yung-Ching Chang, Chan-Chuan Liu, Wan-Ping Chan, Yu-Long Lin, Chun-I Sze, Shiuan-Yeh Chen

**Affiliations:** † Department of Photonics, National Cheng Kung University, Tainan City, 70101, Taiwan; ‡ Department of Cell Biology and Anatomy, National Cheng Kung University, Tainan City 70101, Taiwan; § National Institute of Cancer Research, National Health Research Institutes, Tainan City 70101, Taiwan; ∥ Institute of Basic Medical Sciences, College of Medicine, National Cheng Kung University, Tainan City 70101, Taiwan

**Keywords:** functionalized nanoparticles, Raman tags, anti-EGFR, glioblastoma, GBM, photothermal therapy

## Abstract

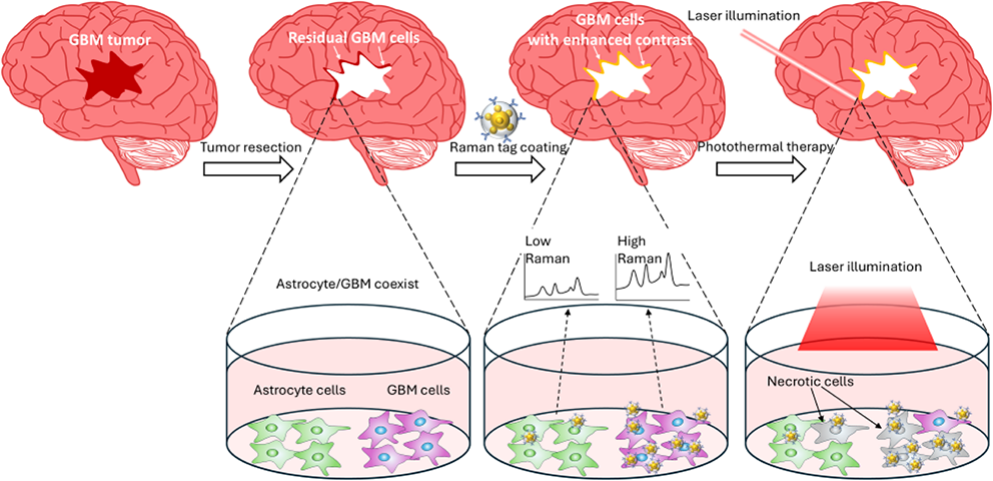

Glioblastoma (GBM) is an aggressive and fatal tumor. The infiltrative spread of GBM cells hinders gross total resection. The residual GBM cells are significantly associated with survival and recurrence. Therefore, a theranostic method that can enhance the contrast between residual GBM and normal astrocyte (AS) cells and selectively eradicate GBM cells is highly desired. In this report, GBM and normal astrocyte cells are both cultured in the same microplate well to imitate a coexistence environment and treated with Raman tags functionalized by anti-EGFR. Compared to AS cells, GBM cells show 25% higher Raman emission, and their cell death rate increases by a factor of 2. These results demonstrate the potential for selective eradication of the residual GBM cells guided by robust Raman signals after the primary GBM surgery.

## 1 Introduction

Glioblastoma (GBM) is the most prevalent malignant brain tumor in adults, constituting 45–50% of all primary malignant brain neoplasms.^1^ According to the World Health Organization’s (WHO) classification criteria, GBM is classified as a central nervous system grade 4 tumor.^1^ The median survival of patients treated by the standard treatment is only 14.6 months,^2^ and the five-year survival rate is 9.8%.^3^ The standard treatment for individuals newly diagnosed with GBM involves undergoing neurosurgery to remove the tumor mass, followed by concurrent radiation therapy and daily doses of temozolomide. Afterward, they typically undergo six cycles of temozolomide.^2^ GBM is notorious for its infiltrative growth, which hinders its precise resection. Infiltrating GBM cells, which escape initial surgical resection and other initial therapies, are the most probable source of local recurrence.^1^ Since the extent of resection and residual volume are associated with survival and recurrence,^4^ for first-line surgery, the method to enhance the contrast between GBM tumors and the surrounding normal tissue and efficiently eliminate the GBM tumor cells is highly desired. In 2017, the US FDA approved fluorescence-guided surgery (FGS) for high-grade gliomas.^5,6^ Patients treated with FGS showed improvement in the completeness of tumor resection compared to conventional white light (65% vs 36%) and higher 6-month progression-free survival.^5^ However, the mixed evidence shows no improvement in overall survival^5^ or some improvement^7^ (17 months vs 10 months) for patients with FGS. In addition, a recent study showed that 5-ALA-induced fluorescence has low accuracy in classifying the fresh tissue samples into GBM or normal tissue.^8^ Therefore, following primary resection, adjuvant therapies to detect and eliminate residual GBM cells remain essential.

In the past decade, exploiting the interaction between nanomaterials and the cell/tissue microenvironment has improved drug delivery, diagnosis, and target therapy for tumor treatments.^9^ Various nanoparticle-mediated treatments for GBM have been proposed and demonstrated.^10,11^ Nanoparticles can serve as imaging, therapeutic, and diagnostic agents.^12−14^ One of the important therapeutic nanoparticles is based on photothermal therapy (PTT).^15−33^ Those PTT nanoparticles with various blood–brain barrier (BBB) crossing strategies are normally delivered to the tumor site through circulation.^21−33^ These strategies include transporter-mediated transcytosis, receptor-mediated transcytosis, cell-mediated transcytosis, the liphophilic pathway, efflux pumps, adsorptive transcytosis, and the paracellular aqueous pathway.^34,35^ On the other hand, the local delivery of the anticancer drugs^36^ or therapeutic nanoparticles^18^ to the resection cavity caused by the first-line GBM surgery can reach residual GBM cells without crossing BBB. However, there are few preclinical efficacy assessments of imaging or therapeutic nanoparticles locally delivered to the tumor resectional cavity where GBM and normal cells coexist. Since EGFR amplification is the most common EGFR alteration in GBM and is observed in 40% of GBM,^1^ the anti-EGFR functionalized nanoparticles can be selectively attached to the GBM cells. A previous study^37^ has used anti-EGFR functionalized nanorods to eradicate two GBM cell lines (U373-MG, 1321N1) and focused on comparing the eradication of the cells without nanorods, with unfunctionalized nanorods, and with functionalized nanorods. In this study, we focus on the selective eradication of GBM/normal astrocytes since the coexistence of GBM/normal astrocytes is expected along the margin of the resectional cavity.

**Figure 1:**
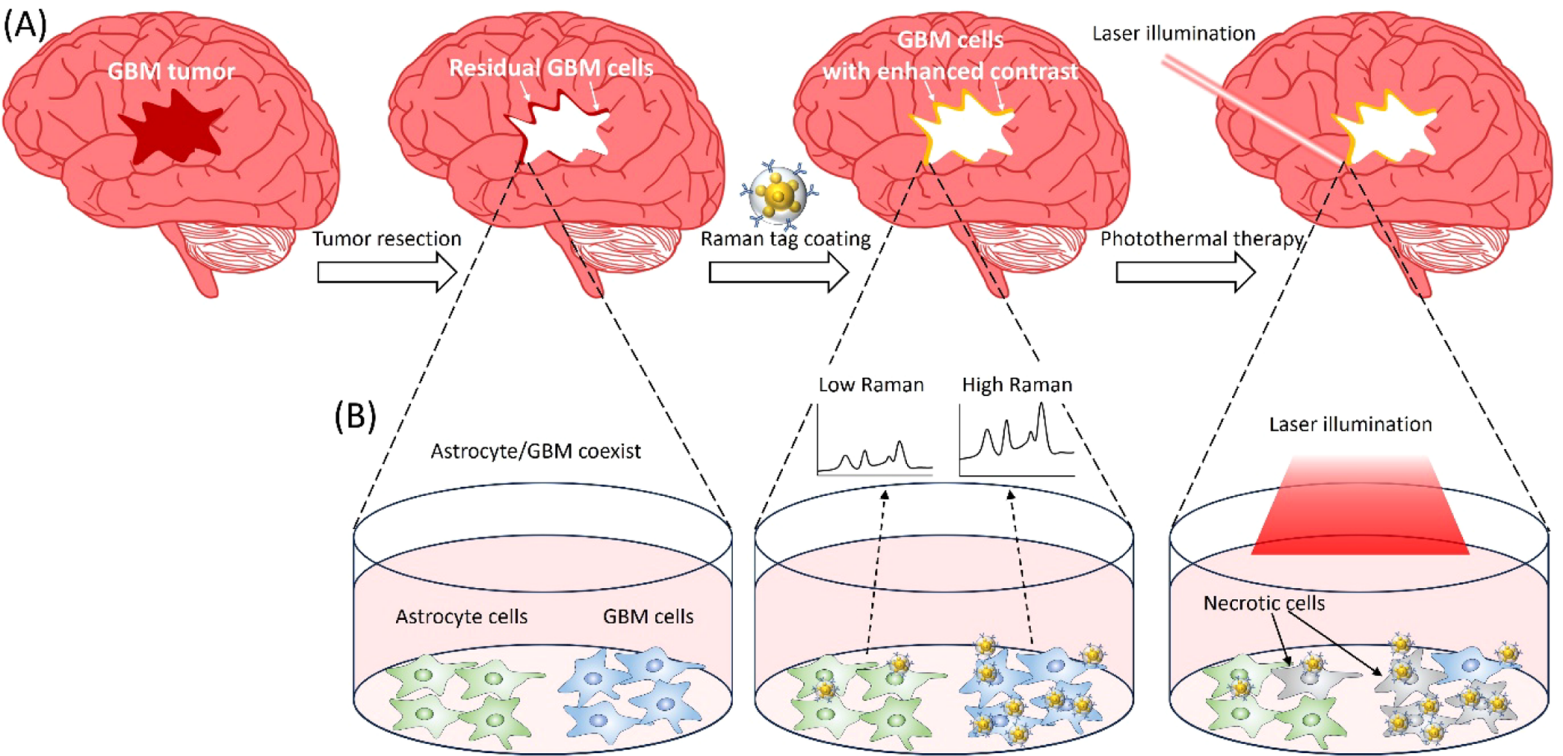
(A) The schematic illustration of the proposed photothermal eradication of residual GBM cells after conventional tumor resection. (B) The corresponding in vitro experiments in this study show that the GBM cells can be selectively eliminated.

**Figure 2:**
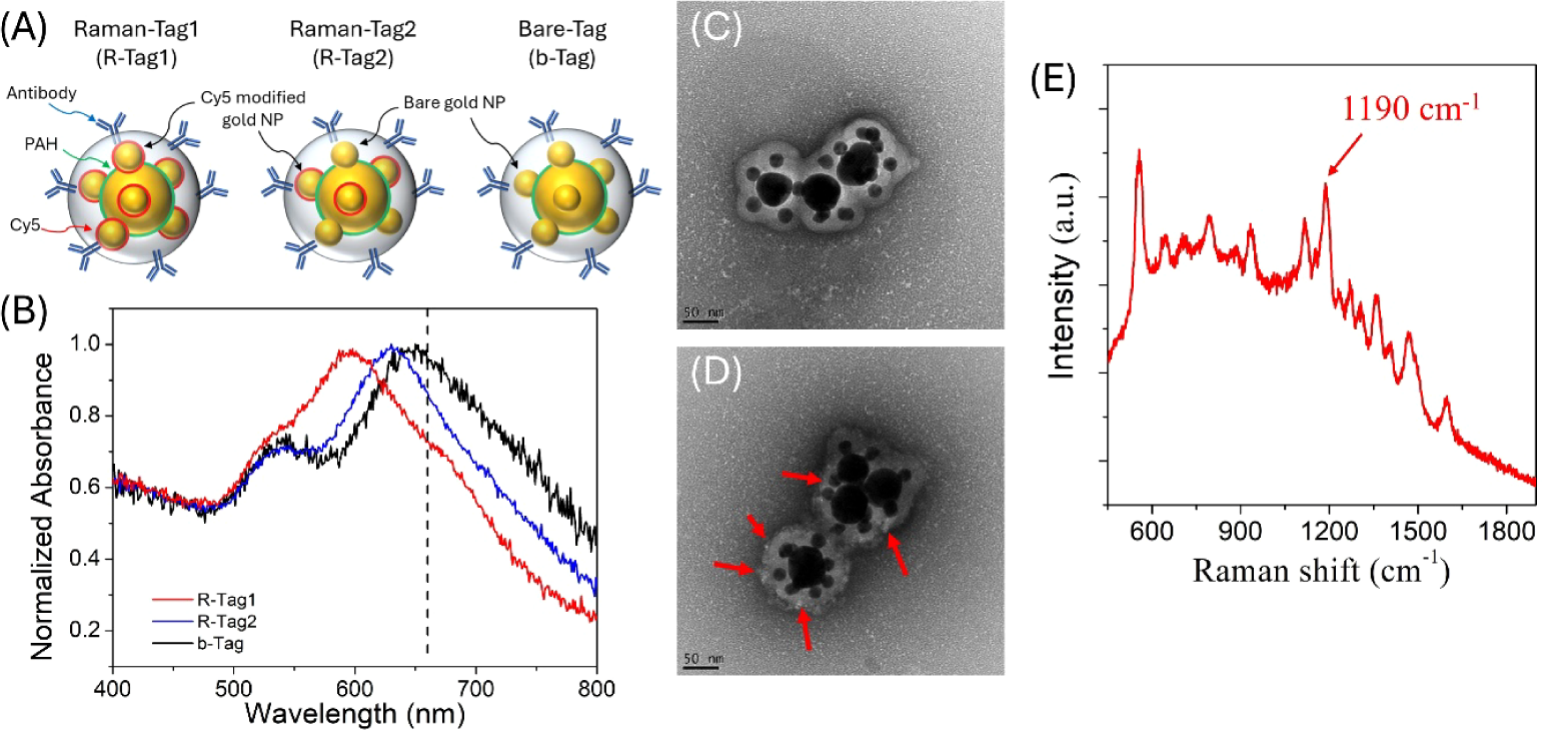
(A) Illustration of b-Tag, R-Tag1, and R-Tag2. (B) The corresponding extinction spectra. Dashed line: wavelength of the laser for photothermal eradication. The TEM images of R-Tag2 before (C) and after (D) antibody coating. (E) Raman signals emitted from R-Tag1.

In this work, Raman tags based on gold nanoparticle assemblies are constructed to provide a stable Raman and photothermal source. Rat GBM cells (CNS-1) and normal rat astrocytes (AS) are both cultured in the same well to imitate the coexistence of residual GBM cells and surrounding astrocytes at the margin of the cavity caused by the primary resection (Figure 1). One bare-Tag (b-Tag) and two Raman tags (R-tag1, R-tag2) are assembled and coated with anti-EFGR to selectively bind to GBM cells, as shown in Figure 2A. The b-Tag is utilized to find the approximate illumination condition for photothermal eradication. R-Tag1 and R-Tag2 are used to acquire the Raman contrast and lead to photothermal eradication simultaneously.

## 2 Results and Discussion

### 2.1 Characterization of Bare Tags and Raman Tags

The tags (Figure 2A) are characterized by three methods. The resonance wavelength of the tags is obtained by the extinction spectrum. As shown in Figure 2B, R-Tag1, R-Tag2, and b-Tag have primary resonance wavelengths at 598, 631, and 647 nm, respectively. This resonance results from the plasmonic coupling between the core and satellite NPs, while the secondary resonance around 540 nm is from the single-particle plasmonic mode. The success of antibody modification is verified by chemical bonds shown in the FTIR spectrum, such as 1541 cm^–1^ and 1644 cm^–1^ for amide II and amide I bonds, respectively. The details are described in our previous report.^38^ The binding of antibodies can also be confirmed directly by observation of the TEM images before and after antibody coating. In Figure 2C, the surface of the silica shell is smooth, while in Figure 2D, it is straightforward to observe negatively stained antibodies (bright spots pointed by red arrows). The distinct peaks of the predefined Raman feature from Cy5 can be observed from the Raman tags (Figure 2E). The intensity of the prominent Raman peak at 1190 cm^–1^ is used to calculate the Raman contrast.

### 2.2 Effectiveness of Photothermal Eradication by B-Tag

The photoinduced eradication of CNS-1 cells can be achieved by choosing the proper conditions, such as tag concentration, incubation time, irradiance, and illumination time. Once the photoinduced eradication of CNS-1 is observed, we must verify whether this effectiveness results from the photothermal effect or the laser-induced toxicity. The impact of intrinsic laser-induced toxicity is verified with CNS-1 treated with no tags. In addition, if the intrinsic laser intensity has low or no effect on cell death, we must clarify that the photothermal eradication results from specific binding or nonspecific binding of the b-tag. Therefore, the CNS-1 cells are also treated with b-Tags coated with anti-EGFR (b-Tag@anti-EGFR) and b-Tags coated with isotype IgG (b-Tag@IgG). The anti-EGFR antibody is a specific antigen-recognizing immunoglobulin G (IgG), while an isotype IgG does not bind the target antigen. Isotype IgG would bind to irrelevant antigen due to nonspecific interactions, such as sticky surfaces, solution condition, and other experimental artifacts.

**Figure 3:**
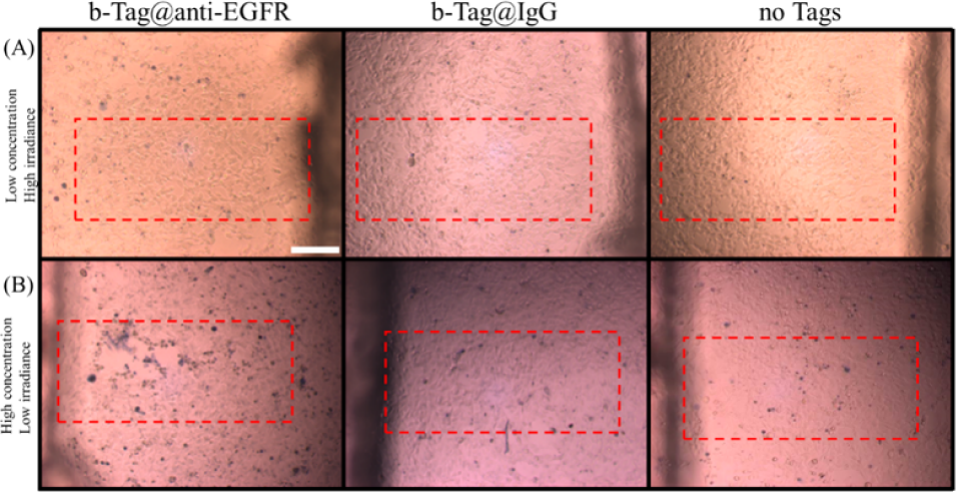
Representative images of CNS-1 cells after photothermal illumination. The laser scanning region is highlighted by the red rectangle. Scale bar: 200 um. The incubation time is 9 h. (A) Exp1: low tag concentration (4.3 pM)/high irradiance (820 W/cm^2^). (B) Exp2: high tag concentration (8.6 pM)/low irradiance (410 W/cm^2^).

For clarity, each of the following experiments is labeled as experiment no. Exp#, and their complete experimental conditions are listed in Table S1. In Exp1, the following parameters are used: incubation times (4.5, 9, and 17 h), tag concentration (4.3 pM), and irradiance (820 W/cm^2^). As shown in Figures 3A and S3, the laser illumination with b-Tag@anti-EGFR, b-Tag@IgG, or no tags does not lead to cell necrosis, no matter what incubation time is adopted.

Then, in Exp2 (Table S1), the particle concentration is increased to 8.6 pM, while the laser power density is decreased to 410 W/cm^2^. As shown in Figures 3B and S4, cell necrosis is only observed from cells labeled with b-Tag@anti-EGFR. The average cell death rate is around ∼50%, while for CNS-1 treated with b-Tag@IgG and no tags, the average death rate is <2%. The low death rate for cells treated with b-Tag@IgG proves nonspecific binding cannot cause sufficient photothermal eradication in Exp2. In addition, for the group without tags, the low death rate indicates that the laser-induced toxicity is insignificant. Only CNS-1 treated with b-Tag@anti-EGFR can cause significant photothermal eradication of GBM cells.

### 2.3 Selectivity of Photothermal Eradication by B-Tag for CNS-1 and AS

**Figure 4:**
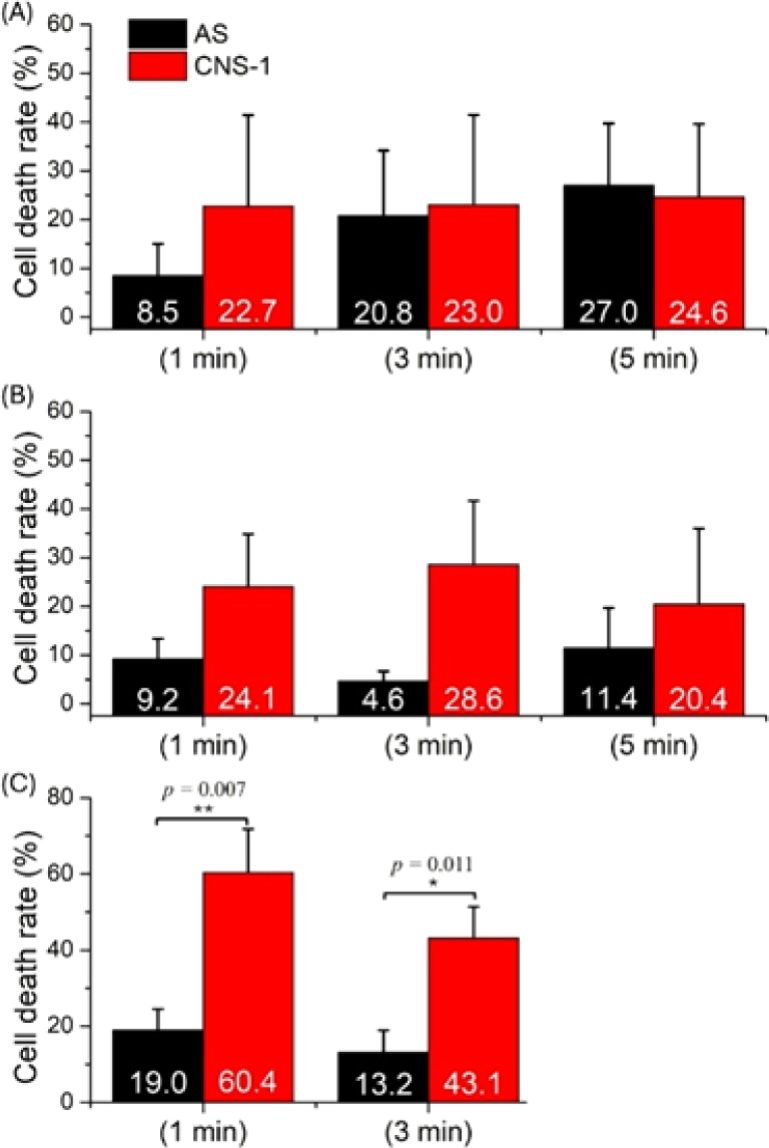
Cell death rate of AS and CNS-1 treated with b-Tag vs illumination time under different tag concentrations and incubation times. (A) 8.6 pM, 9 h. (B) 25 pM, 3 h. (C) 33 pM, 3 h.

Since Exp2 (Table S1) shows the photothermal eradication of CNS-1 cells with b-Tag@anti-EGFR, in Exp3 (Table S1), we apply this condition to see if it can selectively eradicate CNS-1 rather than AS cells. In addition, three lengths of illumination time are also adopted to investigate if it will affect the outcome of photothermal eradication, as shown in Figure 4A (complete cell images in Figure S5). For CNS-1, the death rates of three illumination times are similar (∼20%), while for AS, the death rate is increased with a longer illumination time. Thus, the contrast of the death rate is not apparent for 3 and 5 min illumination but more obvious for 1 min illumination. However, it is not conclusive due to the variation of the cell death rate.

In Exp4 (Table S1), the concentration is further increased to 25 pM, and the incubation time is decreased to 3 h. As shown in Figure 4B (original images in Figure S6), in all three illumination lengths, the death rates of CNS-1 (24.1%, 28.6%, 20.4%) are all higher than those of AS (9.2%, 4.6%, 11.4%). Among them, 1 and 3 min illuminations have higher cell death rate contrast than the 5 min illumination. Although the cell death rate contrast is improved in Exp4, statistical significance for the cell death rate of CNS-1/AS has not yet been achieved.

So far, the approximate conditions for selectively eradicating CNS-1/AS seem to be obtainable. In Exp5 (Table S1), CNS-1 and AS are cocultured in the same well to imitate the coexistence environment at the margin of a resection cavity. The b-Tag concentration is slightly increased to 33 pM. The significant death rate contrast is shown in Figure 4C (original images in Figure S7). Under 1 min illumination, the death rate is 60% in CNS-1, higher than the 19% in AS. Under 3 min illumination, the death rate is 43% in CNS-1, also higher than the 13% in AS. Thus, the conditions in Exp5 can selectively eliminate CNS-1 labeled by b-Tag.

### 2.4 Photothermal Eradication and Raman Contrast of CNS-1/RA by R-Tag1

Since the effectiveness and selectivity of the photothermal eradication by b-Tag are achieved in Exp5, the Raman tag (R-Tag1) replaces the b-Tag in Exp6 to add Raman contrast to CNS-1/AS cells. Observation of both Raman contrast and selective photothermal eradication is expected. The condition in Exp6 (Table S1) is the same as Exp5 except that R-Tag1 is used, and 5 min illumination is included again. However, compared with the cells treated with b-Tag in Figure 4, CNS-1 and AS cells labeled with R-Tag1 scarcely show cell necrosis (data not shown).

Therefore, in Exp7 (Table S1), we increase the reaction time from 3 h to 4/8 h and measure the Raman signals first. The Raman signal is always more prominent in CNS-1, which means that selective adsorption of R-Tag1 occurs. For the 4 h incubation, the average Raman contrast between CNS-1 and AS is 1.46; for the 8-h incubation, the Raman contrast decreases to 1.2. The lower Raman contrast shown in the group with longer incubation (8 h) may result from the adsorption of R-Tag1 due to the nonspecific binding of anti-EGFR to the AS cells. The longer incubation time increases the chance of nonspecific binding. For all 18 observation points, cell necrosis is only observed in two points (data not shown). The prominent Raman contrast indicates the selective adsorption of R-Tag remains effective. However, the failure of the photothermal eradication implies that the photothermal efficiency of R-Tag1 may be substantially lower than that of b-Tag. In addition, the cytotoxicity of Raman tags to GBM and AS cells is also performed in Exp7. Under 4/8 h incubation with Raman tags but without laser illumination, no obvious cell death is observed, which indicates that the Raman tags are nontoxic to GBM and AS cells, as shown in Figure S8.

Under the same tag concentration and illumination time, R-Tag1 fails to reproduce the effectiveness and selectivity of photothermal eradication shown by b-Tag. This failure may result from the considerable blueshift of the resonance frequency from 647 to 598 nm, away from the wavelength of photothermal excitation (660 nm) (Figure 2B). For R-Tag1, the insertion of the Raman reporter, oligonucleotide-modified Cy5, enlarges the gap between the core and satellite nanoparticles and reduces the plasmonic coupling. Thus, the blueshift reflects the reduced coupling. The extinction of R-Tag1 is significantly lower at 660 nm, which may lead to inefficiency of photothermal conversion. Therefore, the efficacy of photothermal eradication by R-Tag1 dramatically deteriorates.

### 2.5 Photothermal Eradication and Raman Contrast of CNS-1/RA by R-Tag2

To recover the photothermal efficiency of Raman tags, a red shift of resonance close to 660 nm is necessary. Therefore, half of the Cy5-functionalized satellite NP is replaced by the bare satellite NP to construct R-Tag2. As shown in Figure 2B, the peak resonance of the extinction of the R-Tag2 is substantially red-shifted to 631 nm.

In Exp8 (Table S1), R-Tag2@anti-EGFR is applied to the CNS-1/AS coexistence environment to observe the cell death rate and Raman contrast. Among nine illuminated regions (6 for 1 min illumination, 3 for 3 min illumination), CNS-1 cells have a higher death rate and Raman signal strength than AS in 8 regions.

**Figure 5:**
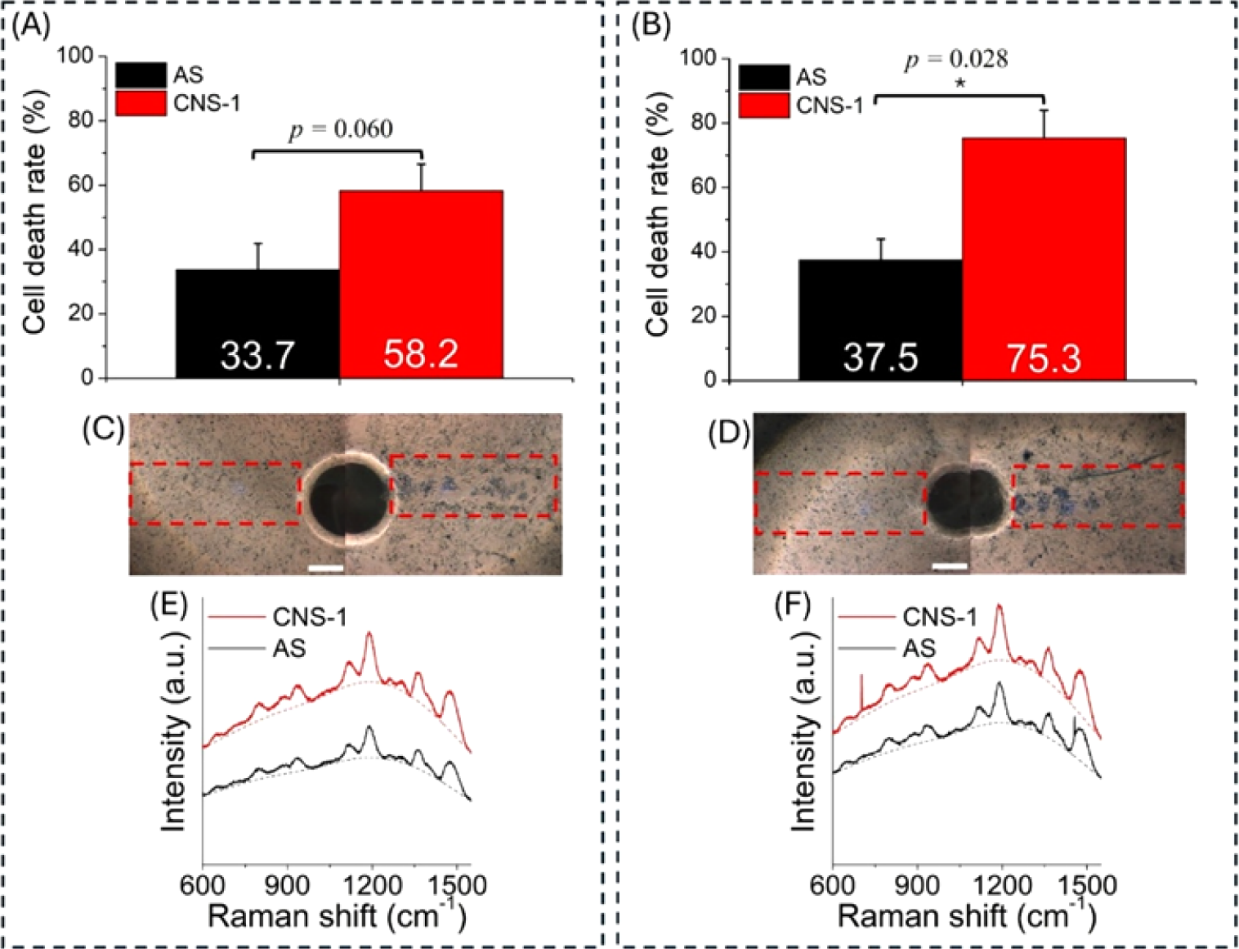
CNS-1/AS cells treated with R-Tag2 under (left) 1 min and (right) 3 min photothermal illumination. (A, B) CNS-1/AS death rate. (C, D) Representative cell images (scale bar: 200 um) and (E, F) the corresponding Raman spectra.

Under 1 min of illumination, the average death rate is 33.7% and 58.2% for AS and CNS-1, respectively (Figure 5A,C). The average death rate contrast is 2.12, and the average Raman contrast is 1.26 (Figure 5E). The correlation coefficient for death rate contrast and Raman contrast is 0.88. Under 3 min illumination, the average death rate is 37.5% and 75.3% for AS and CNS-1, respectively (Figure 5B,D). The average death rate contrast is 2.21, and the average Raman contrast is 1.23 (Figure 5F). The correlation coefficient for death rate contrast and Raman contrast is 0.52.

Interestingly, a similar average death rate contrast is observed under both photothermal illumination lengths (2.12 vs 2.21). The photothermal effect causes a substantially higher death rate in CNS-1 by a factor of 2. Thus, CNS-1/AS cells labeled with R-Tag2 demonstrate Raman contrast and selective photothermal eradication in the coexistence environment.

### 2.6 Discussion

The tag concentration and resonance wavelength of the tags are two critical factors of the effectiveness of photothermal eradication. According to Exp1 and Exp2, a higher concentration leads to the initial efficacy of the photothermal eradication of CNS-1 cells. For the effect of illumination time on GBM cells, under lower tag concentration (8.6 pM in Figure 4A and 25 pM in Figure 4B), the GBM death rate is not sensitive to the illumination time and remains at ∼25%. Under a higher tag concentration (33 pM in Figure 4C and 43 pM in Figure 5), the GBM cell death significantly increases to >40% but is still not sensitive to the illumination time. Furthermore, in Exp8, the similar death rate contrast is observed for 1 and 3 min illumination. Therefore, compared to tag concentration and resonance wavelength, illumination time is probably not the key factor under our experimental conditions.

Exp3 to Exp5 also show that the increasing tag concentration results in better selectivity of photothermal eradication of CNS-1/AS cells. Comparing b-Tag (Exp5) to R-Tag1 (Exp6) indicates that the offset of the resonance wavelength is detrimental to the photothermal performance of the tags. The R-Tag2 in Exp8 demonstrates the selective photothermal eradication of CNS-1 again, even though its extinction is slightly lower than that of the b-Tag. The b-Tag in Exp5 (Figure 4C) has better selectivity of photothermal eradication than R-Tag2 in Exp8 (Figure 5A,B). However, the R-Tag2 can provide Raman and cell death rate contrast simultaneously.

A thinner silica shell could enhance the photothermal conduction of Raman tags. The current 20 nm thickness could be decreased to <5 nm by other silica coating processes such as silicate deposition. The ∼25% Raman contrast can be further enhanced by increasing the brightness of the Raman tags through tuning the resonance of the assembly. Increasing the concentration of the Raman tags might also improve the Raman contrast.

## 3 Conclusion

This work demonstrates the selective photothermal eradication of GBM (CNS-1)/astrocyte (AS) cells when they are cultured in the same well and treated with Raman tags. The cell death rate of CNS-1 is higher than that of AS by a factor of 2. In addition, the Raman signal of the CNS-1 region is 25% higher than that of AS cells. Combining Raman contrast and selective photothermal eradication, this work demonstrates the possibility of eliminating the residual GBM cells around the edge of the resectional cavity after primary surgery. The Raman tags can be further integrated with hydrogels for controlled release. In addition, the portable Raman system can be integrated with a photothermal laser to perform residual GBM cell elimination, guided by Raman signals.

## 4 Methods

### 4.1 Bare and Raman Tag Preparation

In this work, a bare tag (b-Tag) and two Raman tags (R-Tag1, R-Tag2) are all based on a core–satellite assembly (CSA) consisting of a 50 nm gold nanoparticle (NP) as a core surrounded by several 20 nm gold NPs as satellites through positively charged polyelectrolytes (Figure 2A). The b-Tag has no embedded Raman reporters, so its assembly time is 1 day less than that of Raman tags. Thus, b-Tag is used to promptly obtain approximate illumination conditions, including illumination time, power density, tag concentration, etc., for photothermal eradication. The first Raman tag, R-Tag1, has a core particle surrounded by several 20 nm gold NPs coated with oligonucleotide-modified Cy5. Cy5 is the primary Raman reporter (Figure 2E). The oligonucleotide is used to keep the surface charge of the gold NP negative. The second Raman tag, R-Tag2, has the same configuration as R-Tag1 except that the bare 20 nm NP replaces half of the Cy5-coated 20 nm NP to tune the resonance frequency close to the laser excitation to optimize the photothermal effect. The core–satellite assembly is coated with a silica shell and then functionalized with anti-EGFR to selectively bind to GBM cells. The isotype IgG is functionalized for the control group.

To construct b-Tag, 800 μL of citrate-stabilized 50 nm gold nanoparticle suspension (BBI) is diluted by 720 μL of DI water and then mixed with 80 μL of 6 mM poly(allylamine hydrochloride) (PAH). The mixed solution is centrifuged at 2040 *g* for 15 min three times to remove excess PAH. The supernatant is removed for the first two centrifugations, and the same amount of DI water is added. For the third centrifugation, after the supernatant was removed, DI water is added to reach a total volume of 435 μL. This PAH-coated gold NP suspension is extracted using a 1 mL syringe. The 617 μL of 20 nm gold NP suspension (BBI) is prepared in the other 1 mL syringe. The needles of the two syringes are separated by ∼2 mm. The 20 and 50 nm gold NP suspensions are ejected from the tip of the syringe needles and then mixed drop by drop. The total mixing time is around 5 min, and the mixed suspension’s color shifts from red to blue due to the plasmonic resonance coupling between the core and satellite gold NP.

To construct R-Tag1, first, citrate-stabilized 20 nm gold nanoparticles (1.16 nM, BBI solution) are mixed with oligonucleotide-modified Cy5 through the salt-aging method. 125 μL of oligonucleotide-modified Cy5 (2.95 μM) is added to 800 μL of 20 nm AuNP. 1 M of NaCl (1 M) and sodium phosphate buffer (0.1 M) (pH 7) are prepared with a volume equal to eighth of the mixture of gold NP/Cy5-modified oligonucleotide. The 1/8 aliquot of NaCl and sodium phosphate buffer is added every 1 h for the first 8 h, respectively. After 16 h of incubation, the solution of Cy5-modified 20 nm gold NP suspension is centrifuged at 5200 *g* for 20 min three times. After the third removal of the supernatant, DI water is carefully added to maintain the initial concentration of 20 nm gold NP. The 435 μL Cy5-modified 20 nm NP and 617 μL of 50 nm PAH-coated gold NP are mixed to form R-Tag1.

The assembly of R-Tag2 is the same as that of R-Tag1 except that after the last centrifugation of 20 nm NP, the DI water is refilled with DI water to a higher concentration of 2.8 nM. The 126.5 μL of Cy5-modified 20 nm NP and 308.5 μL of citrate-stabilized 20 nm NP are mixed. The 435 μL of 20 nm NP is mixed with the same amount of PAH-modified 50 nm NP through the needle tip of the syringe. After being left to stand for 15 min, 182 μL of DI water is added to obtain 1052 μL of R-Tag2 suspension.

All three tags are coated with a silica shell followed by anti-EGFR. Here, the general processes are described. The detailed steps can be found in our previous report.^38,39^ For the silica coating, APTMS ((3-aminopropyl)trimethoxysilane, Alfa Aesar) is added to produce an anchor layer where silica would grow, followed by the addition of TEOS (tetraethyl orthosilicate, Showa). An initial silica shell is formed by the hydrolysis of TEOS. Then, APTMS is added again to increase the formation of the amino group on the surface of the silica shell. The whole solution is incubated at 35 °C and 800 rpm for 24 h. For anti-EGFR functionalization, the amino group (−NH_2_) is replaced by a carboxyl group (−COOH) by using SA (succinic anhydride, Acros Organics). EDC (*N*-ethyl-*N*’-(3- dimethylaminopropyl)carbodiimide hydrochloride, Sigma-Aldrich) and sulfo-NHS (N-hydroxysulfosuccinimide sodium salt, Sigma-Aldrich) are added to produce an active ester bond. Anti-EGFR can react with the ester bond to form the amide bond. After the functionalization of anti-EFGR, 0.25% BSA is added to the tag suspension to increase the stability.

### 4.2 Cell Preparation

The rat GBM (CNS-1) and rat astrocyte (AS) cells are grown in 3.5 cm culture dishes. The subculture is performed when the population of cells reaches 70% for CNS-1 and 90% for AS. To seed cells in a 96-well microplate, the cells in the culture dish are suspended by trypsin, counted with cell counters, and then diluted with Dulbecco’s Modified Eagle Medium (DMEM) to the working concentration: 5 × 10^4^/mL for CNS-1 and 11.5 × 10^4^/mL for AS. 200 μL of cell suspension is injected into a well of the microplate. Due to the higher cell growth rate of CNS-1, the cell expansion time is 19 h for CNS-1 and 24 h for AS.

### 4.3 GBM and AS Cells Prepared in the Same Microplate Well

120 μL portion of DMEM is added into a well of a 96-well microplate. The PP film coated with PDMS along the edge is inserted into the middle of the well to divide the well into two compartments. 15 μL of AS cell suspension is added into a compartment of the well. Five hours later, 15 μL of CNS-1 suspension is added into the other compartment. After 19 h of incubation, the PP spacer is removed, and then, CNS-1 and AS cells are exposed to the same external condition. For each well, 110 μL of DMEM is extracted, and then, 90 μL of Raman tag suspension is added. The incubation time ranges from 3 to 17 h, depending on the experiment conditions. After incubation, 50 μL of the medium is removed. The whole sample is rinsed with 200 μL of 1× PBS three times. Then, 200 μL of serum-free medium is added to the well for 60 min of incubation. Finally, CNS-1 and AS are ready for Raman acquisition and photothermal illumination in this coexistence environment.

### 4.4 Raman Signals Acquisition

Raman signals from Raman tags attached to CNS-1 and AS cells in the coexistence environment are acquired by an inverted microscope with a 632.8 nm HeNe laser and a spectrometer (grating is 1200 l/mm with a slit size of 300 μm). The laser is focused by a 10× objective lens, concentrating the laser light into a point ∼200 μm in diameter. A marker is engraved at the backside of the center of the target well. The marker is used as a positional reference point. For each side of the cells, the Raman signal is acquired sequentially from 5 points equally spaced (250 μm) along a straight line. After Raman acquisition, the 96-well microplate is moved back to the incubator for 30 min to stabilize the cells.

### 4.5 Photothermal Setup and Procedure

After Raman acquisition, the whole microplate is moved to a custom photothermal setup. The 660 nm CW laser beam is focused to a spot of ∼200 μm in diameter. This work adopts two irradiance settings (410 and 820 W/cm^2^). Three illumination times (1, 3, and 5 min) for each illuminated spot are utilized. The spacing of each spot is 150 μm.

### 4.6 Cell Death Rate Calculation

To evaluate the efficacy of photothermal treatment, the cell death rate of illuminated cells must be defined. After the photothermal treatment, Trypan blue staining is performed to determine cell death. The dead cells become blue. The whole blue area is integrated by ImageJ and then divided by the illuminated area to obtain the cell death rate.

### 4.7 TEM Characterization of Anti-EGFR Functionalized Raman Tags

Anti-EGFR functionalized Raman tags are characterized by TEM, and the sample is processed by negative staining. The carbon film of a TEM grid (CF200-Cu, EMS) is hydrophilized by a hydrophilizing system (JEOL HDT-400). 4 μL tag suspension is deposited on the TEM grid. After 50 s, the excess suspension is removed by filter paper. The grid is rinsed with 4 μL of DDI water, and then, the excess water is removed. Then, 0.1 M uranyl acetate is dropped onto the grid, kept for 50 s, and then removed by filter paper. After the grid is dried, the sample is ready for observation under TEM (JEOL, JEM-1400).

## Supporting Information Available

S1: experimental conditions for photothermal eradication experiments from Exp1 to Exp8; S2: the photothermal profiles and photothermal images of the tags; S3–S7: the complete set of cell images acquired after photothermal eradication in Exp1 to Exp5; S8: the cytotoxicity test of Raman tags in Exp7; S9: the complete set of cell images acquired after photothermal eradication in Exp8 (PDF)
