# Supplementary material for: Selective Photothermal Eradication of Glioblastoma Cells Coexisting with Astrocytes by Anti-EGFR-Coated Raman Tags
Source: ACS Appl Bio Mater. 2025 Mar 18;8(4):3119–26. doi: 10.1021/acsabm.4c01986 (PMC12015947; doi:10.1021/acsabm.4c01986)
Supplement: Supplementary file 1 — mt4c01986_si_001.pdf [file mt4c01986_si_001.pdf]

Supporting Information

Selective Photothermal Eradication of Glioblastoma Cells Coexisting with Astrocytes by anti-EGFR Coated Raman Tags

YUNG-CHING CHANG[1], CHAN-CHUAN LIU[2,3,4], WAN-PING CHAN[1], YU-LONG LIN[1], CHUN-I SZE[2,4], SHIUAN-YEH CHEN[1*]

[1] Dept. of Photonics, National Cheng Kung University, Tainan City, Taiwan 70101
[2] Dept. of Cell Biology and Anatomy, National Cheng Kung University, Tainan City, Taiwan 70101
[3] National Institute of Cancer Research, National Health Research Institutes, Tainan City, Taiwan 70101
[4] Institute of Basic Medical Sciences, College of Medicine, National Cheng Kung University, Tainan City, Taiwan 70101
*sychen72@ncku.edu.tw

S1: Experimental conditions for photothermal eradication experiments from Exp1 to Exp8.

Table S1 Experimental conditions for each photothermal eradication experiment.

	Cell	Tag	Coexistence	Tag Concen. (pM)	Incubation time (hr)	Irradiance (W/cm^2)	Illumin. time (min)	Figure
Exp1	CNS-1	b-Tag@Ab<br>b-Tag@IgG<br>No Tags		4.3	4.5/9/17	820	5	Fig. 3(A)<br>Fig S3
Exp2	CNS-1	b-Tag@Ab<br>b-Tag@IgG<br>No Tags		8.6	9	410	5	Fig. 3(B)<br>Fig. S4
Exp3	CNS-1<br>AS	b-Tag@Ab		8.6	9	410	1,3,5	Fig. 4(A)<br>Fig. S5
Exp4	CNS-1<br>AS	b-Tag@Ab		25	3	410	1,3,5	Fig. 4(B)<br>Fig. S6
Exp5	CNS-1<br>AS	b-Tag@Ab	✓	33	3	410	1,3	Fig. 4(C)<br>Fig. S7
Exp6	CNS-1<br>AS	R-Tag1@Ab	✓	33	3	410	1,3,5	
Exp7	CNS-1<br>AS	R-Tag1@Ab	✓	33	4/8	410	1,3, no laser	Fig. S8
Exp8	CNS-1<br>AS	R-Tag2@Ab	✓	43	8	410	1,3	Fig. 5<br>Fig. S9

S2: The photothermal profiles (Fig. S2-1) and photothermal images (Fig. S2-2) of the tags. Two tag concentrations and two peak irradiances are adopted.

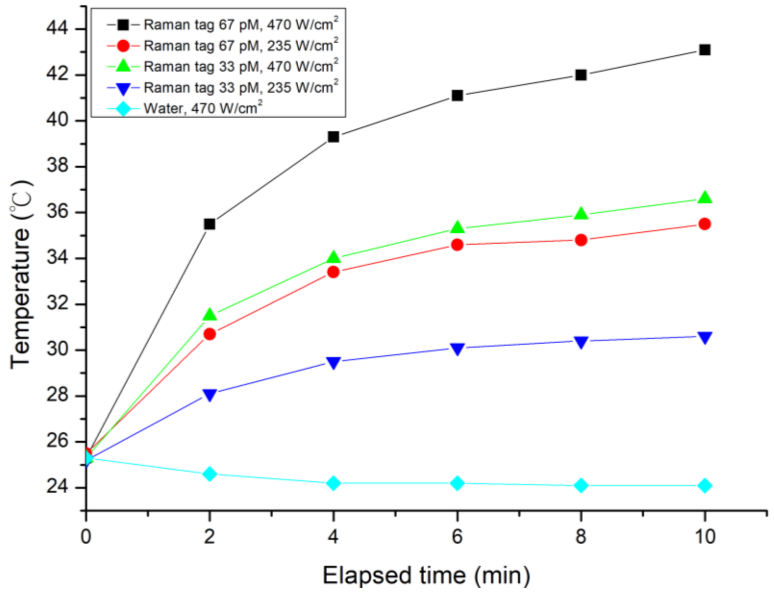

Fig. S2-1 The photothermal profile of tags under two concentrations (67 pM, 33 pM) and peak irradiances (470W/cm^2, 235 W/cm^2).

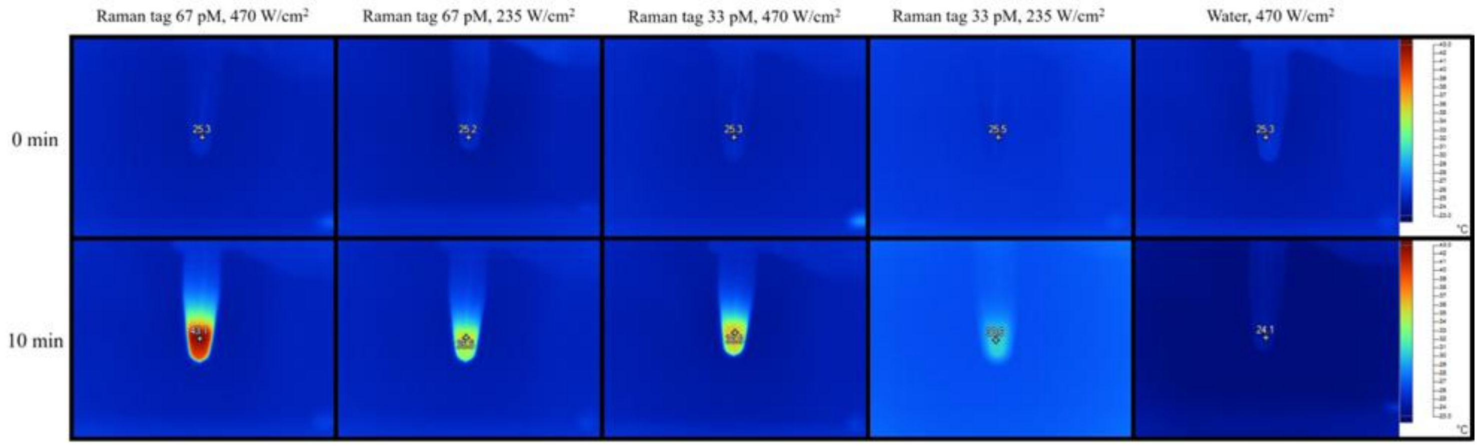

Fig. S2-2 The photothermal images of 100 uL of tag suspension in a microcentrifuge tube.

S3: The complete set of cell images acquired after photothermal eradication in Exp1.

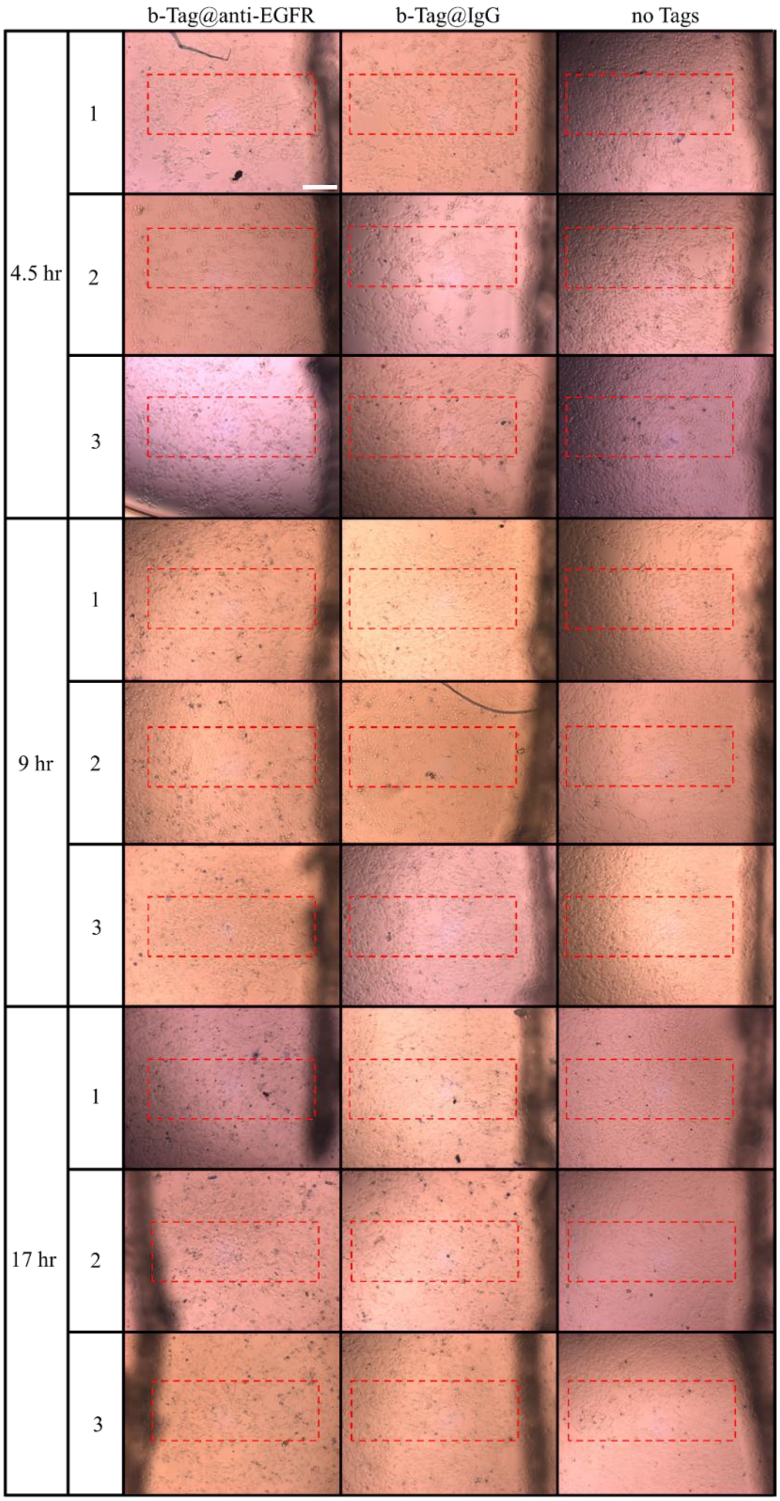

Fig. S3 The complete set of cell images acquired after photothermal eradication in Exp1 (Scale bar: 200 um).

S4: The complete set of cell images acquired after photothermal eradication in Exp2.

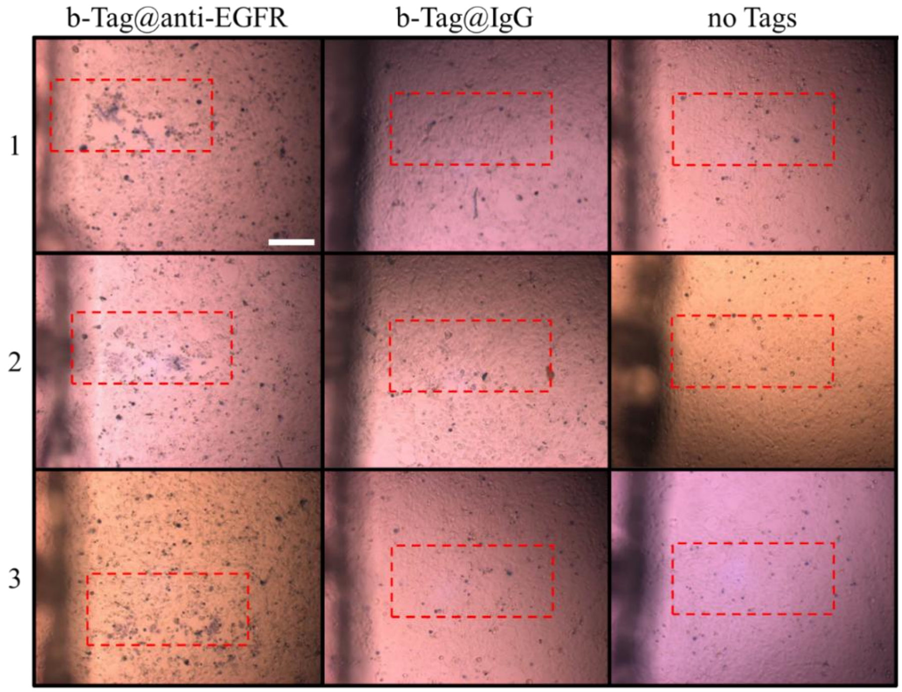

Fig. S4 The complete set of cell images acquired after photothermal eradication in Exp2 (Scale bar: 200 um).

.

S5: The complete set of cell images acquired after photothermal eradication in Exp3.

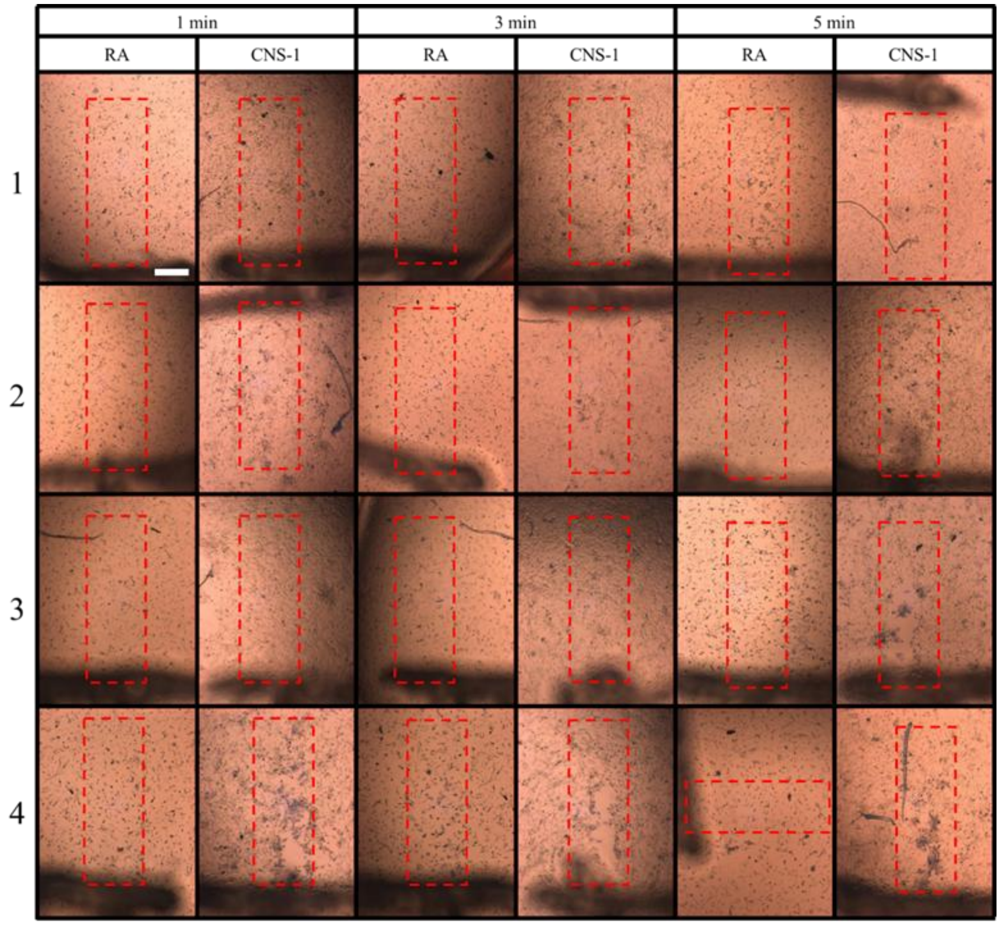

Fig. S5 The complete set of cell images acquired after photothermal eradication in Exp3 (Scale bar: 200 um). The corresponding statistical chart is shown in Fig. 4(A).

S6: The complete set of cell images acquired after photothermal eradication in Exp4.

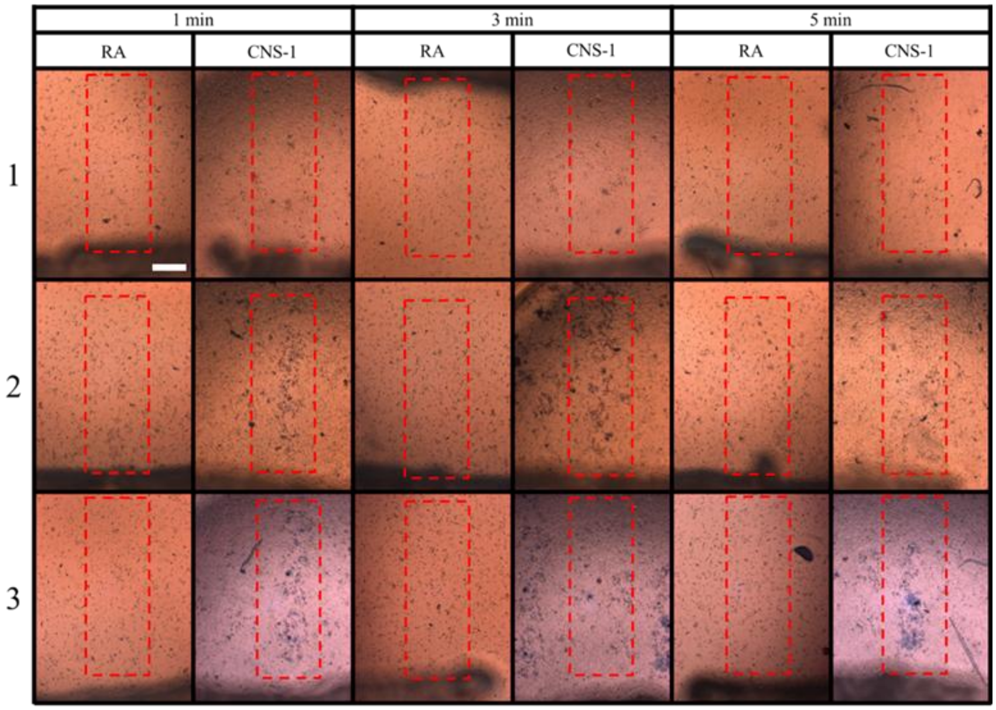

Fig. S6 The complete set of cell images acquired after photothermal eradication in Exp4 (Scale bar: 200 um). The corresponding statistical chart is shown in Fig. 4(B).

S7: The complete set of cell images acquired after photothermal eradication in Exp5.

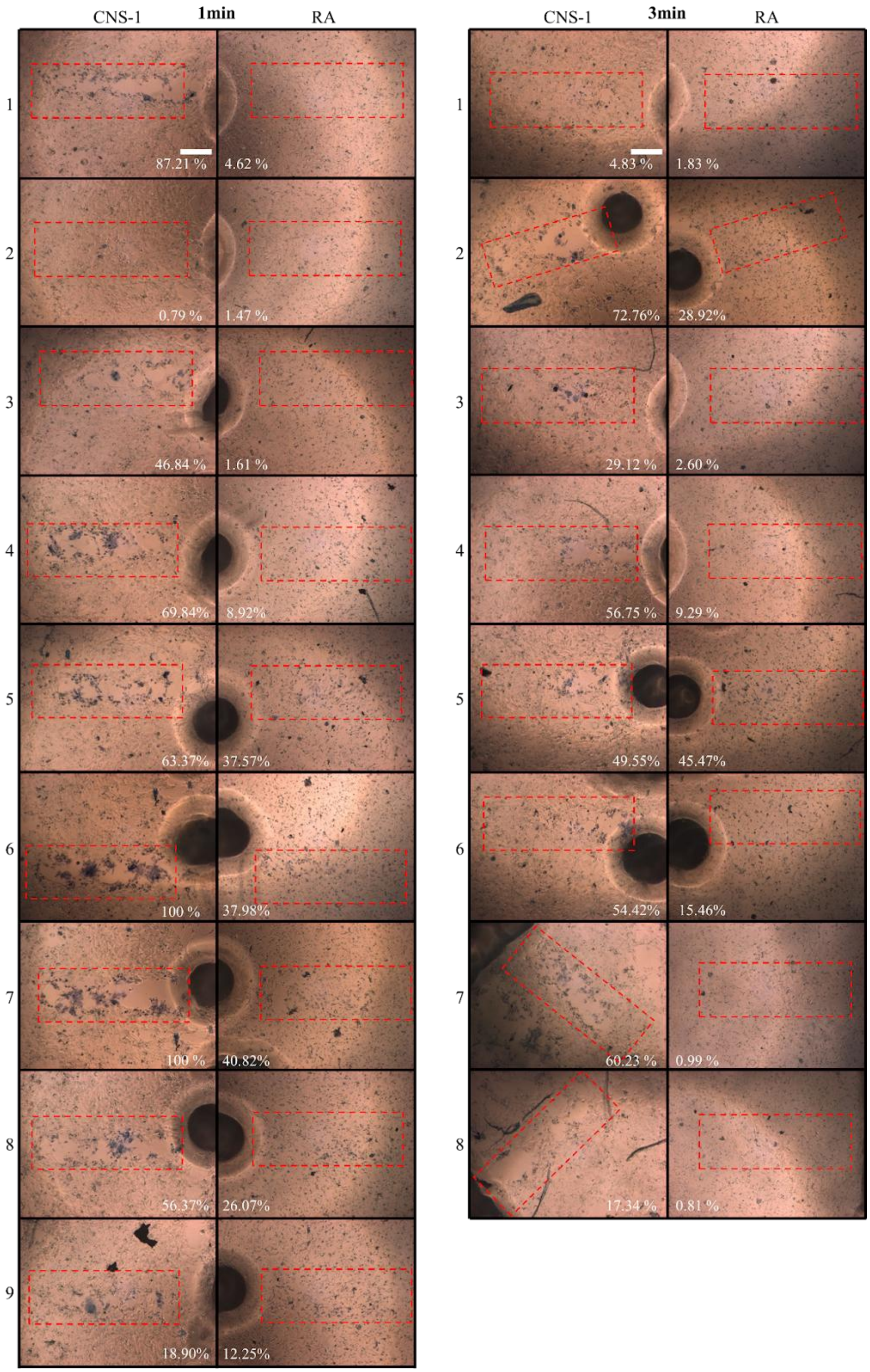

Fig. S7 The complete set of cell images acquired after photothermal eradication in Exp5 (Scale bar: 200 um). The corresponding statistical chart is shown in Fig. 4(C).

S8: The cytotoxicity test of Raman tags in Exp7.

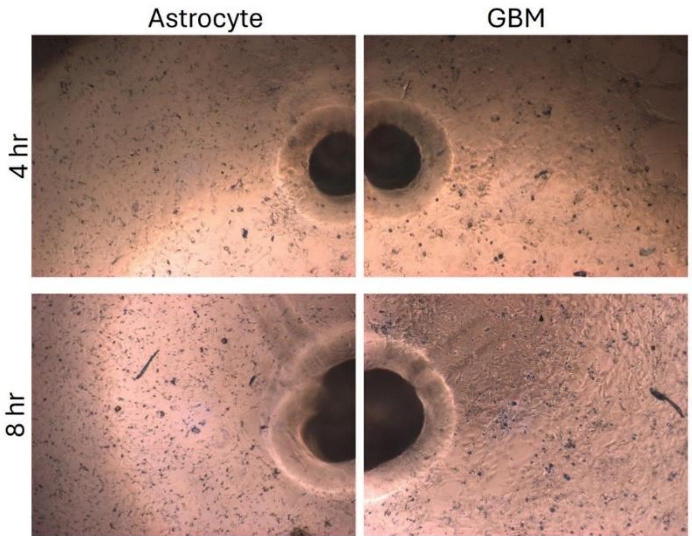

Fig. S8 AS and GBM cells incubated in 33 pM of R-Tag1 for 4 and 8 hr without laser illumination.

S9: The complete set of cell images acquired after photothermal eradication in Exp8.

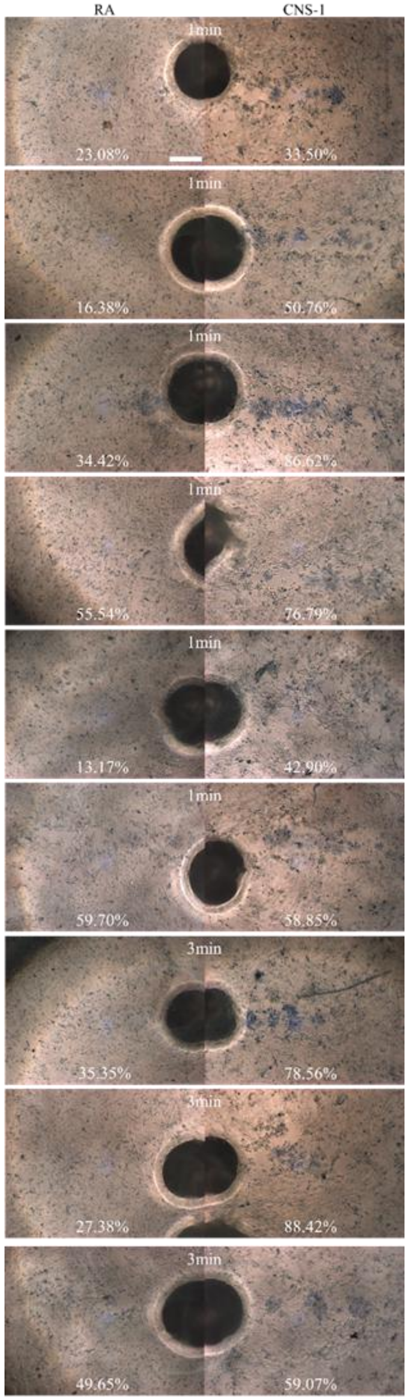

Fig. S9 The complete set of cell images acquired after photothermal eradication in Exp8 (Scale bar: 200 um). The corresponding statistical chart is shown in Fig. 5(A)(B).
